# Inhibition of APE1 Expression Enhances the Antitumor Activity of Olaparib in Triple-Negative Breast Cancer

**DOI:** 10.1155/2022/6048017

**Published:** 2022-04-13

**Authors:** Dan Jian, Xue-Mei Li, Nan Dai, Dan-Dan Liang, Gang Zhang, Cheng-Yi Mao, Dong Wang, Guan-Bin Song, Meng-Xia Li, Hao Luo

**Affiliations:** ^1^Cancer Center, Daping Hospital, Army Medical University, Chongqing 400042, China; ^2^Department of Thyroid and Breast Surgery, Daping Hospital, Army Medical University, Chongqing 400042, China; ^3^Department of Pathology, Daping Hospital, Army Medical University, Chongqing 400042, China; ^4^College of Bioengineering, Key Lab of Biorheological Science and Technology, Ministry of Education, Chongqing University, Chongqing 400030, China

## Abstract

Triple-negative breast cancer (TNBC) is a highly aggressive subtype of breast cancer that is prone to recurrence and metastasis. Because of the lack of expression of estrogen receptor (ER) and progesterone receptor (PR) and human epidermal growth factor receptor 2 (HER2) in TNBC, treatment methods are greatly limited. In this study, the proliferation inhibition and apoptosis-inducing effects of PARP1 inhibitors in TNBC breast cancer cells and in vivo xenograft animal models were examined to investigate the molecular role of APE1 in PARP1-targeted therapy. In TNBC patients, the expression of APE1 and PARP1 were positively correlated, and high expression of APE1 and PARP1 was associated with poor survival of TNBC. Our results indicated that knockdown APE1 could increase the sensitivity of olaparib in the treatment of TNBC. In conclusion, the results of this study will not only clarify the molecular role of APE1 in PARP1-targeted therapy for TNBC but also provide a theoretical basis for the future clinical application of targeting APE1 and PARP1 in the treatment of refractory TNBC.

## 1. Introduction

Triple-negative breast cancer (TNBC) is a highly aggressive subtype of breast cancer that is prone to recurrence and metastasis [1], accounting for about 20% of breast cancer patients [2, 3]. Due to the lack of expression of estrogen receptor (ER) and progesterone receptor (PR) and human epidermal growth factor receptor 2 (HER2) in TNBC, treatment methods such as endocrine therapy and molecular targeted therapy are greatly limited [4]. In addition, up to 70% and 23% of TNBC show histological characteristics of genomic BRCA1 and BRCA2 mutations, respectively [5–7]. At the ASCO 2009 Annual Meeting, data from an important study reported that inhibiting the activity of poly (ADP-ribose) polymerase-1 (PARP1), a key enzyme related to DNA repair, can selectively induce apoptosis in cells with BRCA1/2 gene mutations, implying that molecular therapy targeting PARP1 may become a new potential treatment for TNBC. The current phase II and phase III clinical studies also indicated that PARP1 inhibitors combined with radiotherapy and chemotherapy can significantly improve the clinical benefits in overall survival (OS) and progression-free survival (PFS) [1, 8, 9]. However, the objective response rate of PARP1 inhibitor alone for TNBC was only 41%, which may have problems such as insensitivity or resistance. Different from traditional radiochemotherapy, targeted therapy may trigger drug resistance by activating alternative pathways. Therefore, it is important to clarify the activated alternative pathways that cause drug resistance to provide further molecular targets for subsequent drug development or combination therapy.

Apurinic/apyrimidinic endonuclease/redox factor-1 (APE1/Ref-1, hereinafter referred to as APE1) is a multifunctional protein with DNA damage repair and redox reduction. In addition, APE1 is one of the important members of the base excision repair (BER) pathway [10]. In the DNA damage repair pathway, both PARP1 and APE1 participate in the BER repair pathway. Therefore, in the targeted therapy of PARP1, APE1 may compensate for the impaired activity of PARP1 in the BER repair pathway [11, 12], thereby affecting the therapeutic effect. In other words, APE1 may be related to the drug resistance of PARP1-targeted therapy for TNBC patients, and it may also indicate that APE1 may be a new potential target for TNBC.

In this study, the proliferation inhibition and apoptosis-inducing effects of PARP1 inhibitors in TNBC breast cancer cells and in vivo xenograft animal models were examined to investigate the molecular role of APE1 in PARP1-targeted therapy. In addition to knock down the expression of APE1 to investigate the DNA repair activity of PARP1, PARP1 inhibitors were further combined to explore the combined therapeutic effect on TNBC. The results of this study will not only clarify the molecular role of APE1 in PARP1-targeted therapy for TNBC but also provide a theoretical basis for the future clinical application of targeting APE1 and PARP1 in the treatment of refractory TNBC.

## 2. Materials and Methods

### 2.1. Cell Culture

MCF10A, MDA-MB-231, MDA-MB-436, and MCF-7 cell lines were obtained from American Type Culture Collection (ATCC, Manassas, VA, USA) and Chinese Academy Sciences Cell Bank of Type Culture Collection (Shanghai, China), respectively. All cells were cultured in Dulbecco's modified Eagle medium (DMEM; Invitrogen, Carlsbad, CA, USA) supplemented with 10% fetal bovine serum (FBS; HyClone, Logan, UT) at 37°C in humidified atmosphere containing 5% CO_2_.

### 2.2. Clinic Data Analysis

The total of 60 patients with TNBC was enrolled in this study at Daping Hospital, Army Medical University (Chongqing, China), between July 2019 and July 2021. The eligible patients were enrolled according to the following criteria: patients did not receive previous chemotherapy or radiotherapy and did not have other malignancy in 5 years before this study; patients with spinal compression, pregnancy, lactation, serious infection, or impairment of organ functions were excluded. All patients were diagnosed with TNBC according to WHO classification. Immunohistochemical detection of these tissue samples is also observed (Table 1).

### 2.3. TCGA Data Analysis

To analyze the expression of APE1 and PARP1 in TNBC, the data in TCGA were downloaded, and a total of 114 paracancerous tissues and 123 cancer tissues were obtained with clear indication. The differences were analyzed separately, and the edgeR package in the R language was applied. The difference conditions were log|FC|>1, *P* < 0.05.

### 2.4. RT-qPCR

Briefly, total RNA in cells were extracted by TRIzol reagent (Thermo Fisher Scientific, Waltham, MA, USA). For APE1, using the PrimeScript RT reagent kit (Takara, Shiga, Japan), each total RNA sample (1 *μ*g) was subjected to reverse transcription reaction to obtain the cDNA template, and the detail was performed as described by Li et al. [13]. Sequences of the double-stranded siRNAs are antisense (5′-GUCUGGUACGACUGGAGUACC-3′, 5′-UACUCCAGUCGUACCAGACCU-3′) and nonsense (5′-CCAUGAGGUCAGCAUGGUCUG-3′,5′-GACCAUGCUGACCUCAUGGAA-3′).

### 2.5. Measurement of Cell Viability and Cell Invasion

Cell viability was determined by the Cell Counting Kit-8 (Biosharp, Hefei, Anhui, China). Briefly, cells were seeded in 96-well culture plates at a density of 5000 cells per well. After overnight incubation, the cells were incubated with CCK-8 reagent for indicated times, and the cell viability was measured using according to the manufacturer's instructions.

### 2.6. Western Blot Analysis, Immunohistochemistry (IHC), and Immunofluorescence (IF) Assay

Western blot analysis, IHC, and IF assays were performed as described previously [10, 13–15]. Cells were lysed using RIPA lysis buffer (Sigma-Aldrich). In this study, antibodies against APE1 (1 : 500), PARP1(1 : 1000), BCL2(1 : 500), *γ*-H2AX (1 : 500), and Ki-67(1 : 500) as well as HRP or FITC-linked mouse IgG were purchased from Cell Signaling Technology (Danvers, MA, USA). Histone-H3(1 : 500) and *β*-actin (1 : 500) antibodies were obtained from Proteintech (Rosemont, IL, USA). Hematoxylin and DAPI were obtained from Sigma-Aldrich.

### 2.7. Detection of Apoptotic Cells

Cell apoptosis was using the FITC Annexin V apoptosis detection kit (BD biosciences, San Jose, CA, USA). After the cells were incubated with indicated drugs for indicated times, the cells were collected and incubated with a FITC/Annexin V and propidium iodide (PI) according to the manufacturer's instructions. Apoptotic cells were further detected and analyzed by flow cytometry. Flow cytometry apoptosis assays were performed using the FITC Annexin V apoptosis detection kit (BD biosciences, San Jose, CA, USA), according to the manufacturer's instructions.

### 2.8. Measurement of DNA Damage by Comet Assay

The comet assay (single-cell gel electrophoresis) was performed as described by Li et al. [13]. Briefly, the cells were mixed with 0.5% low melting point agarose and then placed in a horizontal gel electrophoresis chamber covered with freshly prepared electrophoresis buffer. After electrophoresis, the cells were stained with ethidium bromide and observed under fluorescence microscopy. Comet was analyzed using Komet 5.5 software.

### 2.9. Subcutaneous Xenograft Mouse Model

For the subcutaneous xenograft model, MDA-MB-231 (2 × 10^6^ in 0.1 mL) were subcutaneously injected into 6-week-old female BALB/c nude mice. When the injected tumors grew to about 50 mm^3^ in size, the mice were randomly divided into four groups, and the mice were administrated with indicated drugs according to the classified groups. The mice were administrated subcutaneously with olaparib (1.5 mg/kg) or vehicle control every 3 days for 4 weeks. The bodyweight and the tumor size of each mouse were measured once a week. The tumor volume was calculated by the formula: *V* = (*W*^2^ × L)/2. In this study, the nude mice were provided by the Experimental Animal Center of Chongqing Medical University and cultured in SPF grade animal laboratory. All animal experiments were conducted in accordance with the Chongqing University Policy for the Care and Use of Laboratory Animals, and the animal protocol was approved by the Animal Experimentation Ethics Committee of the Chongqing Medical University.

### 2.10. Statistical Analysis

All statistical analyses were performed using SAS statistical software version 6.12 (SAS institute). Data were presented as the mean ± standard deviation (SD). The differences between the groups were analyzed by Student's *t*-test or one-way analysis of variance (ANOVA). Survival analysis was performed using Kaplan–Meier, and overall survival (OS) was used to assess patient survival. A *p* value of less than 0.05 was considered statistically significant.

## 3. Results

### 3.1. High Expression of APE1 and PARP1 Is Associated with Poor Prognosis of TNBC Patients

**Figure 1:**
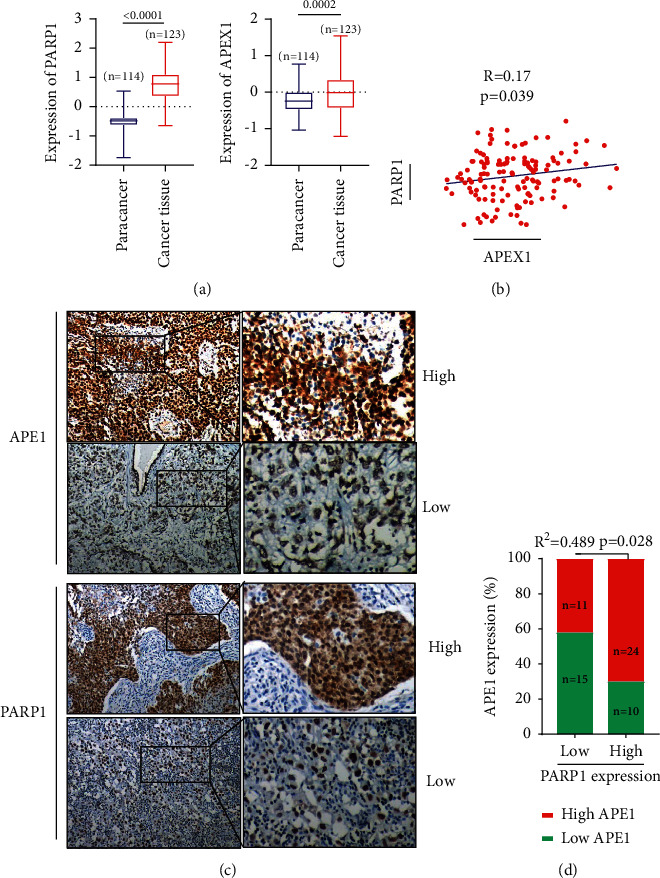
The correlation between APE1 and PARP1 expression in TNBC patients. (a) The TCGA datasets examined the expression of APE1 and PARP1 in TNBC. (b) The TCGA datasets analyzed the correlation of APE1 and PARP1 in 134 TNBC. (c) The 60 clinical pathological tissue samples tested by immunohistochemical to analyze the correlation of APE1 and PARP1 expression. (d) The histogram of APE1 and PARP1 expression.

**Figure 2:**
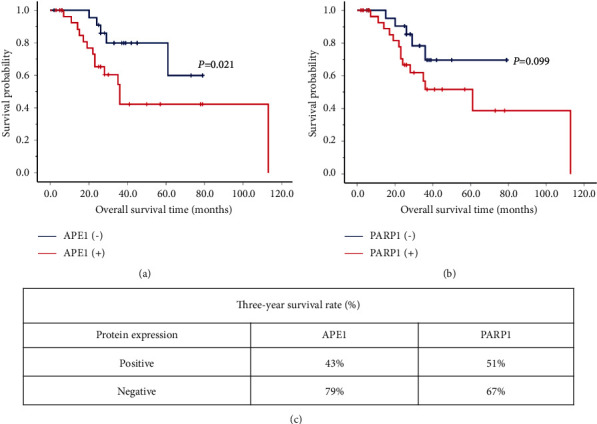
High expression of APE1 and PARP1 are associated with poor prognosis of TNBC patients (a) The Kaplan–Meier survival analyzed the relationship between the expression of APE1 and TNBC patient survival. (b) The Kaplan–Meier survival analyzed the relationship between the expression of PARP1and TNBC patients' survival. (c) The influence of the expression of APE1 and PARP1 on the three-year survival rate of TNBC patients.

Since APE1 and PARP1 are important genes involved in DNA damage repair, the TCGA datasets were used to examine the expression of APE1 and PARP1 in TNBC. As shown in Figures 1(a) and 1(b), the expression levels of APE1 and PARP1 were significantly increased in TNBC. The results were further confirmed by immunohistochemical analysis of 60 clinical pathological tissue samples, and the results showed that the APE1 expression was positively correlated and also PARP1 expression (Figures 1(c) and 1(d)). PARP1 is associated with axillary lymph node metastasis, *P*=0.009. However, APE1 is associated with tissue differentiation, *P*=0.027 (Table 1). In addition, survival analysis showed that patients with higher expression of APE1 (Figure 2(a)) or PARP1 (Figure 2(b)) were associated with poor prognosis. The 3-year survival rates of patients with high expression of APE1 and PARP1 were 43% and 51%, respectively, while the 3-year survival rates of patients with low expression of APE1 and PARP1 were 73% and 67%, respectively (Figure 2(c)). Therefore, we speculate that simultaneous inhibition of APE1 expression may enhance the antitumor activity of PARP1 inhibitors against TNBC.

**Figure 3:**
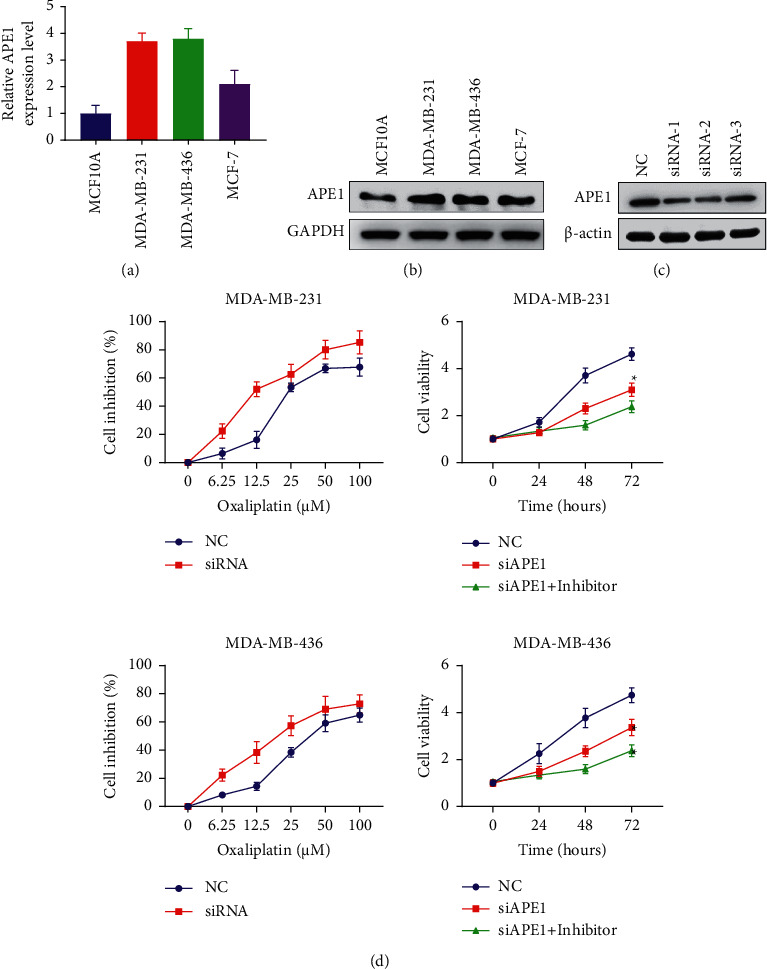
The selection of experimental cells and detection of cell growth. (a) RT-PCR examined the expression levels of APE1 in MCF10A, MDA-MB-231, MDA-MB-436, and MCF-7 cell lines. (b) Western blotting examined the expression levels of APE1 in MCF10A, MDA-MB-231, MDA-MB-436, and MCF-7 cell lines. (c) Western blotting examined the expression levels of APE1 after transfection of siRNA. (d) CCK-8 examined the effect of siAPE1 and olaparib on cell growth in MDA-MB-231 and MDA-MB-436 cell lines.

**Figure 4:**
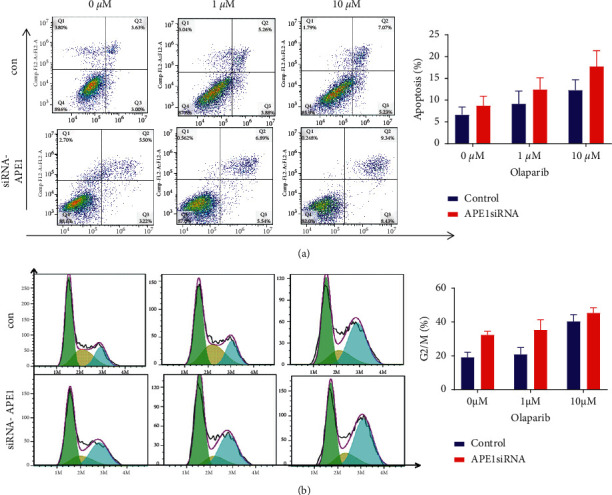
Inhibition of APE1 expression increases the apoptosis and cell cycle arrest of TNBC cells. (a) Flow cytometry examined the effect of siAPE1 and olaparib on cell apoptosis in MDA-MB-231 and MDA-MB-436 cell lines. (b) Flow cytometry examined the effect of siAPE1 and olaparib on cell cycle arrest in MDA-MB-231 and MDA-MB-436 cell lines.

Knockdown of APE1 expression increases the sensitivity of TNBC cells to olaparib to select suitable cell lines. RT-PCR and Western blotting were used to examine the expression levels of APE1 in MCF10A, MDA-MB-231, MDA-MB-436, and MCF-7 cell lines. As shown in Figures 3(a) and 3(b), APE1 was highly expressed in MDA-MB-231 and MDA-MB-436 cell lines. Therefore, these two cell lines were used for subsequent in vitro experiments. When the expression of APE1 in MDA-MB-231 and MDA-MB-436 cells was knockdown by siRNA technology, the sensitivity of these cells to olaparib also increased. As shown in Figure 3(c), olaparib treatment further reduced the cell growth rate of MDA-MB-231 and MDA-MB-436. In addition, APE1 knockdown combined with olaparib treatment further promotes cell apoptosis (Figure 4(a)) in the TNBC cells and cause cell cycle arrest in the G2/M phase (Figure 4(b)).

### 3.2. Combination of APE1 Inhibition and Olaparib Treatment Significantly Reduces Tumor Growth in the Xenograft Mouse Model

**Figure 5:**
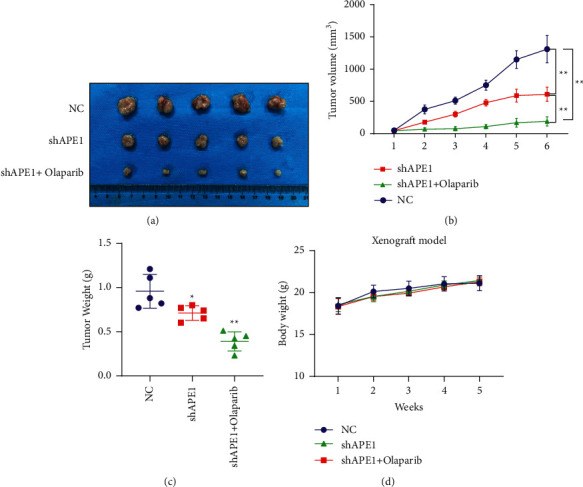
Combination of APE1 inhibition and olaparib treatment significantly suppress tumor growth in the xenograft mouse model. (a) Xenograft figure. (b) Tumor volume in xenografts. MDA-MB-231 cells were transfected with the shAPE1 or treated with the PARP1 inhibitor olaparib. (c) Xenograft weight. (d) The mouse bodyweight.

**Figure 6:**
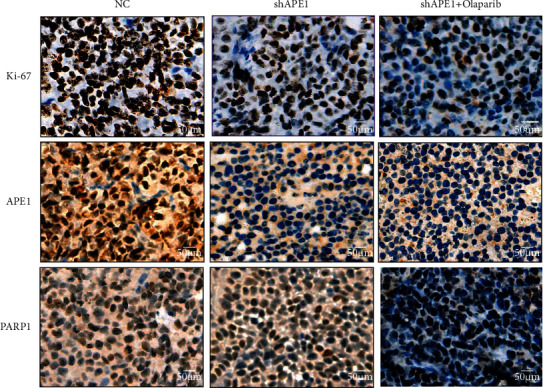
Immunohistochemistry assays of Ki-67, APE1, and PARP1expression in vivo (200×).

Our xenograft animal model in vivo also supported that the combination of siAPE1 and olaparib treatment significantly reduced the tumor volume (Figure 5(a) and 5(b)) and tumor weight (Figure 5(c)) than siAPE1 alone. We did not find significant bodyweight loss (Figure 5(d)). Furthermore, IHC experiments showed that the proliferation index Ki-67 level decreased in the combination of siAPE1 and olaparib treatment (Figure 6). Taken together, these results suggest that inhibition of APE1 expression not only increased the sensitivity to olaparib but also combination of APE1 inhibition and olaparib treatment can suppress tumor growth in vivo.

**Figure 7:**
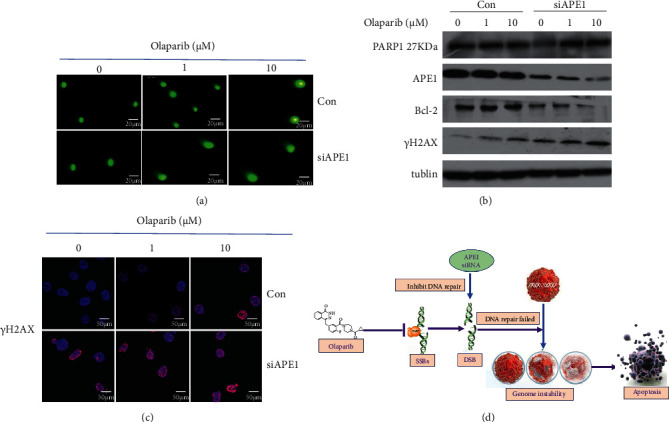
Inhibition of APE1 expression promotes DNA damage caused by PARP1 inhibitors. (a) Comet assay showed that suppression of APE1 significantly stimulated radiation-induced DNA damage compared to control in MDA-MB-231 cells. (b) Western blotting showed that inhibition of APE1 expression can further increase the expression of *γ*H2AX and regulate the expression of apoptosis-related protein Bcl2 and Bax under olaparib treatment. (c) Immunofluorescence analysis of *γ*H2AX levels further confirmed that combination of APE1 inhibition and olaparib treatment promotes DNA damage. (d) A schematic regulatory mechanism showing that inhibition of APE1 expression enhances the antitumor activity of olaparib.

Inhibition of APE1 expression promotes DNA damage caused by olaparib. Next, comet assay was used to detect and quantify the formation of DNA strand breaks in individual cell. As shown in Figure 7(a), inhibition of APE1 expression significantly increases the degree of DNA damage caused by olaparib treatment. In addition, inhibition of APE1 expression can further increase the expression of *γ*H2AX and downregulate the expression of BCL2 under olaparib treatment (Figure 7(b)). Immunofluorescence analysis of *γ*H2AX levels further confirmed that combination of APE1 inhibition and olaparib treatment promotes DNA damage (Figure 7(c)).

## 4. Discussion

TNBC, an independent clinicopathological subtype of breast cancer that does not express ER, PR, and HER2, is clinically characterized with high malignancy, strong invasiveness, and poor prognosis [16]. Studies have shown that TNBC is more likely to harbor a germline BRCA1 gene mutation, which leads to weakened DNA double-strand break repair and activates the PARP1 pathway to compensate for repair activities [16]. However, when the BRCA1/2-mediated DNA damage repair pathway is further impaired, the biological function of PARP1 in DNA double-strand break repair becomes important. Therefore, several studies suggested targeting PARP1 may be a potential therapeutic molecular approach for the treatment of TNBC [17, 18]. Although olaparib, a new PARP1 inhibitor developed by AstraZeneca, was approved by the FDA in 2018 for the treatment of breast cancer patients with hereditary BRCA gene mutations, the toxicity and treatment resistance of olaparib still exist. In this study, we performed TCGA analysis and verified with clinical samples; our results showed that PARP1 was highly expressed in TNBC tissues, and the high PARP1 expression is associated with poor survival. These results also supported that PARP1 plays an important role in the occurrence and development of TNBC [19]. In addition, our results also found that the high expression of APE1 is closely associated with the high expression of PARP1 in TNBC patients. In vitro and in vivo experiments have also confirmed that knocking down the expression of APE1 to reduce DNA double-strand repair of tumor cells can enhance the efficacy of olaparib on TNBC. In addition, inhibition of APE1 expression in TNBC further enhanced olaparib-mediated cell apoptosis and cell cycle arrest in the G_2_/M phase. In vivo xenograft animal experiments also confirmed that the combination of APE1 knockdown and olaparib treatment can more significantly inhibit tumor growth compared to APE1 knockdown alone and olaparib treatment alone. Therefore, our results indicated that APE1 is a therapeutic target to increase the sensitivity of olaparib in the treatment of TNBC.

This study also explored the potential mechanism of APE1 inhibition for improving the anti-TNBC activity of olaparib. Through the detection of DNA damage, APE1 knockdown combined with olaparib treatment can further increase the DNA damage of tumor cells, leading to the upregulation of the DNA damage marker *γ*H2AX.

Our findings have certain preclinical significance for the future clinical treatment of TNBC, a strategy to simultaneously target APE1 and PARP1 in TNBC. Our previous study has found that AT101 has an inhibitory effect on APE1 activity [20, 21]. Importantly, AT101 inhibitor has completed phase 1 clinical trials and showed satisfactory results in TNBC treatment [22, 23]. Thus, in addition to olaparib, targeting APE1 by the AT101 inhibitor may also be another feasible alternative for the treatment of TNBC.

## 5. Conclusion

In TNBC patients, the expression of APE1 and PARP1 was positively correlated, and high expression of APE1 and PARP1 was associated with poor survival of TNBC. APE1 is a potential new therapeutic target in the treatment of TNBC with olaparib because both APE1 and PARP1 play an important role in DNA damage repair. Therefore, blocking the APE1 and PARP1 signaling pathways may be expected to become a new treatment strategy for TNBC.

## Figures and Tables

**Table 1:** The relationship between PARP1 and APE1 and clinicopathological parameters of TNBC patients.

	PARP1		*P* value	APE1		*P* value
	Positive	Negative		Positive	Negative	
Age (mean)	48.699	52.012		51.023	48.921	
Age			0.998			0.596
＜50	20	15		19	16	
≥50	14	11		16	9	
Tumor volume			0.768			0.204
≤2	5	6		4	7	
2–5	21	15		24	12	
≥5	7	4		6	5	
Axillary lymph node metastasis			0.009^*∗*^			0.190
Positive	23	8		21	10	
Negative	11	18		14	15	
Clinical stage			0.307			0.857
I	2	5		3	4	
II	19	15		20	14	
III	7	3		6	4	
IV	6	3		5	3	
Tissue differentiation			0.504			0.027^*∗*^
I	1	2		1	2	
II	12	12		10	14	
III	21	12		24	9	

^*∗*^ *P* < 0.05.

## Data Availability

The data used to support this study are included within the article.
